# Targeting Micrometastases: The Effect of Heterogeneous Radionuclide Distribution on Tumor Control Probability

**DOI:** 10.2967/jnumed.117.207308

**Published:** 2019-02

**Authors:** Nadia Falzone, Boon Quan Lee, Sarah Able, Javian Malcolm, Samantha Terry, Yasir Alayed, Katherine A. Vallis

**Affiliations:** 1 CRUK/MRC Oxford Institute for Radiation Oncology, Oxford University, Oxford, United Kingdom; and; 2 Imaging Chemistry and Biology, King’s College London, London, United Kingdom

**Keywords:** Auger-electrons, MC modeling, 3D spheroids, dosimetry, TCP

## Abstract

The spatial distribution of radiopharmaceuticals that emit short-range high linear-energy-transfer electrons greatly affects absorbed dose and biological effectiveness. The purpose of this study was to investigate the effect of heterogeneous radionuclide distribution on tumor control probability (TCP) in a micrometastasis model. **Methods:** The cancer cell lines MDA-MB-468, SQ20B, and 231-H2N were grown as spheroids to represent micrometastases. The intracellular distribution of a representative radiopeptide (^111^In-labeled epidermal growth factor) and radioimmunotherapeutic (^111^In-labeled trastuzumab) was determined in cell internalization experiments. The intratumoral distribution was evaluated by microautoradiography of spheroids. γH2AX staining was performed on spheroid sections to correlate DNA damage with radionuclide distribution. Experimental surviving fractions were obtained using clonogenic assays. A random close-packed algorithm, which models the random packing behavior of cells and reflects variation in the radii of cells and nuclei, was used to simulate 3-dimensional spheroids. Calculated survival fractions were generated using an iterative modeling method based on Monte Carlo–determined absorbed dose with the PENELOPE code and were compared with experimental surviving fraction. Radiobiologic parameters deduced from experimental results and Monte Carlo simulations were used to predict the TCP for a 3-dimensional spheroid model. **Results:** Calculated survival fractions agreed well with experimental data, particularly when an increased value for relative biological effectiveness was applied to self-dose deposited by sources located in the nucleus and when radiobiologic parameters were adjusted to account for dose protraction. Only in MDA-MB-468 spheroids treated with ^111^In-epidermal growth factor was a TCP of more than 0.5 achieved, indicating that for this cell type the radiopeptide would be curative when targeting micrometastases. This ability is attributed to the relative radiosensitivity of MDA-MB-468 cells, high nuclear uptake of the radiopeptide, and uniform distribution of radioactivity throughout the spheroid. **Conclusion:** It is imperative to include biologic endpoints when evaluating the distribution of radionuclides in models emulating micrometastatic disease. The spatial distribution of radioactivity is a clear determinant of biological effect and TCP as demonstrated in this study.

The predicted relative biological effectiveness of targeted radionuclide therapy is often extrapolated from that of external-beam radiotherapy. It is assumed that the biologic response to targeted radionuclide therapy will be similar to that of external-beam radiotherapy when an equivalent absorbed radiation dose is delivered to the tumor as a whole. However, the biologic response to targeted radionuclide therapy varies substantially with differences in expression of the molecular target, which in turn results in spatial nonuniformity of radioactivity and energy deposition and thus absorbed dose (Gy) at the whole-tumor, cellular, and subcellular levels. It is postulated that Auger electron (AE)–emitting radionuclides that bind or intercalate into DNA are ideal for targeted radionuclide therapy of single cells, limited-volume disseminated cancer, and micrometastases (1,2). This hypothesis is based on the high linear-energy transfer (4–26 keV/μm) of low-energy AEs, which deposit energy within a few cubic nanometers of the decay site (1,3). The localized absorption of electrons results in complex irreparable DNA damage (4). This feature endows AE-emitting targeted radionuclide therapy agents with an enhanced relative biological effectiveness when compared with α- or β-emitting radionuclides.

To advance our understanding of the radiobiology of AE-emitting therapeutics and to test the hypothesis that they are ideally suited to the treatment of micrometastases, appropriate biologic models that reflect the in vivo 3-dimensional (3D) architecture and physiology of tumors are required. Also, a dosimetric approach that accounts for both the intratumoral and the intracellular distribution of radioactivity is necessary (5). Multicellular tumor spheroids are excellent models of micrometastases because they reflect the cellular heterogeneity, physiologic gradients (6,7), and resistance to therapy of small tumors (8). By combining experimental data detailing the dose distribution in individual cells and in whole spheroids with Monte Carlo (MC) modeling, radiobiologic quantities such as tumor control probability (TCP), which relates the fate of individual cells to a macroscopic outcome, can be calculated (9,10). Several MC studies focusing on the charge transport of AEs in volumes representing small cell clusters have explored whether the dose deposited would be sufficient to elicit a tumoricidal effect (11,12). However, to date only a few studies have attempted to validate MC simulations that predict the efficacy of AE emitters with experimental observations (13–15). In this study, the efficacy of radiolabeled peptide- and antibody-based constructs was evaluated in spheroids generated from 3 cancer cell lines. ^111^In-DTPA-human epidermal growth factor (hereafter ^111^In-EGF), which causes selective radiotoxicity in EGF receptor (EGFR)–overexpressing cells, was used as a representative radiopeptide, and ^111^In-DTPA-trastuzumab (hereafter ^111^In-Tz), which binds human EGFR-2 (HER2/neu), was used as a representative radioimmunotherapeutic (16,17). Data derived from cell internalization and spheroid microautoradiography experiments were used to build realistic in silico MC models. These models were used to predict TCP and were validated against experimentally derived values.

## MATERIALS AND METHODS

### 3D Culture of Spheroids

The human breast cancer cell lines MDA-MB-468 (1.3 × 10^6^ EGFR per cell; HER2-low) and 231-H2N (0.2 × 10^6^ EGFR, 6.1 × 10^5^ HER2 per cell) and a human head and neck squamous carcinoma cell line, SQ20B (1.0 × 10^6^ EGFR per cell, HER2-low) (16,18), were grown in Dulbecco modified Eagle medium (Sigma-Aldrich) supplemented with 10% fetal bovine serum (Invitrogen), penicillin (100 units/mL), and streptomycin (100 μg/mL) (Sigma-Aldrich) at 37°C in 5% CO_2_. MDA-MB-468 and SQ20B cells were obtained from American Type Culture Collection, and 231-H2N cells were obtained from Robert Kerbel at Sunnybrook Health Sciences Centre. Spheroids were generated using the InSphero hanging-droplet method. MDA-MB-468 (10,000 cells per well), 231-H2N (40,000 cells per well in InSphero 3D culture medium), and SQ20B (20,000 cells per well) were seeded into GravityPLUS plates (InSphero AG). Spheroids were transferred to GravityTRAP plates on day 4.

### Microautoradiography and Immunofluorescence

^111^In-EGF and ^111^In-Tz were synthesized as previously described (16,19). Spheroids were exposed to ^111^In-EGF (8 MBq/μg; 40 nM) or ^111^In-Tz (6 MBq/μg; 10 nM) for 1 or 24 h and then fixed for 30 min (4% paraformaldehyde), washed in phosphate-buffered saline, and placed in a cryomold (Agar Scientific) containing Tissue Tek O.C.T. (Electron Microscopy Sciences). Samples were flash-frozen and stored at −80°C. Samples were sliced into 8-μm-thick sections, thaw-mounted on SuperFrost Plus microscopy slides (VWR International), and dried at room temperature for at least 30 min before being coated with Kodak NTB emulsion (VWR International). Slides were stored in a light-tight box for 24 h at 2°C–8°C until development (Kodak D-19; Sigma-Aldrich) and fixation (Kodak polymer RT, 1:4; Sigma-Aldrich). γH2AX staining was performed as previously described (18) on sequential spheroid sections to evaluate DNA damage.

### Subcellular Distribution of Radioactivity

Cells (1 × 10^6^ cells/mL) were exposed to ^111^In-EGF (8 MBq/μg; 40 nM) or ^111^In-Tz (6 MBq/μg; 10 nM) for 1 or 24 h. Unbound, membrane-bound, cytoplasmic, and nuclear fractions were collected as previously described (16) using a subcellular protein fractionation kit (Thermo Fischer). Purity of fractions was confirmed by Western blot analysis of known membrane, cytoplasmic, and nuclear proteins: β-integrin, α-tubulin, and H2AX. The amount of radioactivity in each fraction was measured by a γ-counter (PerkinElmer Wizard 1470).

### Cell Size Measurement

Cells were spun from a Cytospin (Thermo Fisher Scientific) centrifuge onto slides and fixed (4% paraformaldehyde; Sigma-Aldrich) for 15 min at 37°C. After being washed with phosphate-buffered saline, the cells were covered in a membrane-specific stain, wheat germ agglutinin (5.0 μg/mL) conjugate (WGA-Alexa Flour-488; Thermo Fisher Scientific), for 10 min followed by 4′,6-diamidino-2-phenylindole staining of cell nuclei. The cells were washed in phosphate-buffered saline and mounted with Vectashield mounting medium (Vector Laboratories) before microscopy (TCS SP2 microscope; Leica Microsystems). Cell and nucleus radii were taken as the average of 30 stained cells.

### MC Simulation

To represent 3D spheroids in silico, an event-driven molecular dynamics algorithm (20) for a system of monodisperse spheric random close-packed (RCP) cells was used (21,22). Event-by-event simulation was performed using the MC code PENELOPE (23) and the complete electron spectrum of ^111^In based on the unabridged nuclear decay data (*BrIccEmis*) (24). The photon absorbed fraction was negligible and therefore omitted (5). In total, 1 × 10^8^ primary particles were simulated. Simulations were performed in liquid water, and the spatial distribution of radioactivity within a spheroid was based on the microautoradiography results. Two scenarios were considered (reflecting microautoradiography staining): the first being uniform distribution of radioactivity throughout the spheroid and the second being peripheral accumulation. The radioactivity in each cell of the MC model was informed by experimental subcellular internalization data. Absorbed fractions (*S* values), derived from dose-point kernels of an ^111^In point source in liquid water using terminology described previously (1), were calculated for contributions from a source cell to its nucleus (N←Cs), the cytoplasm to its nucleus (N←Cy), and the nucleus to itself (N←N). The cross dose was derived from the difference between the self-dose and simulated total dose (25). *S* values were calculated for single cells, monolayers, and cell clusters, with the last two using the RCP algorithm.

### Survival Fraction and TCP Calculation

Clonogenic survival assays were performed on monolayers of MDA-MB-468, SQ20B, and 231-H2N cells treated with ^111^In-EGF (8 MBq) or ^111^In-Tz (6 MBq) as previously described (16). The radiosensitivity parameters, α and β, were determined for each cell line after ^137^Cs irradiation (1 Gy/min) by fitting the average surviving fraction from 3 independent experiments using least-squares regression by the linear quadratic model. α- and β-values were, respectively, 0.46 and 0.003 for MDA-MB-468, 0.20 and 0.001 for SQ20B, and 0.12 and 0.06 for 231-H2N cells.

The survival probability of an individual cell, *SP*_*i*_*,* follows the linear-quadratic model (9):

Eq. 1 $$SP_{i}=e^{-\left(\alpha D_{i}+\beta GD_{i}^{2}\right)},$$

where *D*_*i*_ is the absorbed dose (Gy) of an individual cell, α and β are determined from ^137^Cs irradiation, and *G* is the Lea-Catcheside factor, which accounts for radiation damage repair (additional information can be found in the supplemental materials, which are available at http://jnm.snmjournals.org (26–29)). Dose was calculated according to the MIRD formulation (30), where *D*_*i*_ is taken as the product of accumulated activity *Ã* in each cell compartment and its associated *S* value, with τ being the target (nucleus) and σ the source (i.e., N, Cy, or Cs):


Eq. 2 $$D_{i}=\sum_{i}{\tilde{A}}_{\sigma_{i}}\times S\left(\tau \leftarrow \sigma_{i}\right).$$

For a population of *N*_*c*_ identical clonogenic cells that receive a variable absorbed dose, *D*_*i*_, the calculated surviving fraction, *SF*_*cal*_, of the population is


Eq. 3 $$SF_{cal}=\frac{\sum_{i=1}^{N_{c}}SP_{i}}{N_{c}}$$

Thus, the TCP of a 3D spheroid, which is defined as the probability of killing all cells within the spheroid, is given as


Eq. 4 $$\text{TCP}=\prod_{i=1}^{N_{c}}\left(1-SP_{i}\right)$$

### Statistical Analysis

Statistical analyses were performed using Prism (version 5; GraphPad Software, Inc.). Internalization and clonogenic surviving fraction data were analyzed using 2-way ANOVA with the Sidak multiple-comparison test and the Tukey multiple-comparison test, respectively. Statistical significance is reported as a *P* value of less than 0.05.

## RESULTS

### 3D Spheroid Model

**FIGURE 1.**
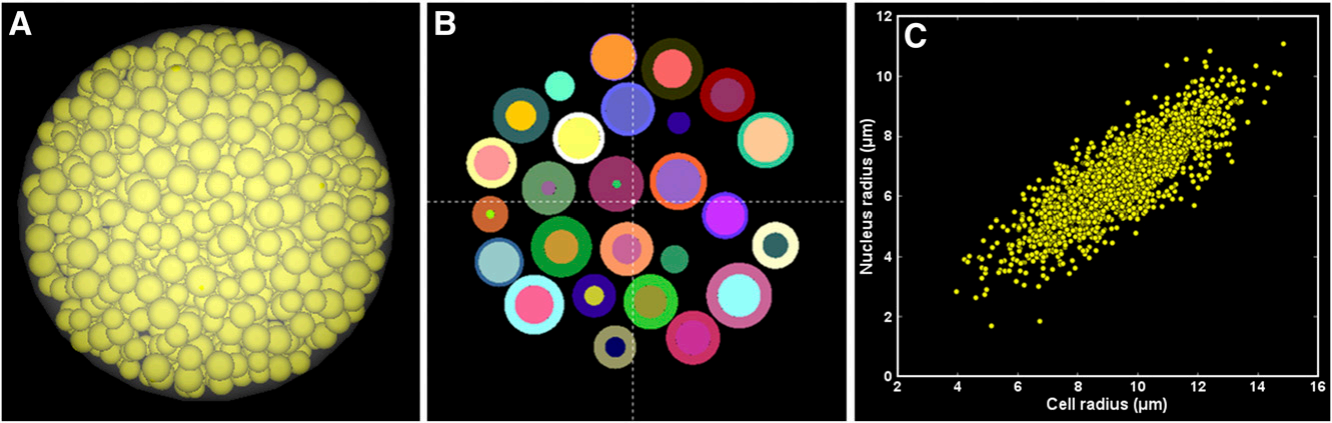
(A) Packing of monodisperse spheric RCP cells according to cell size distribution of MDA-MB-468 cell line. (B) Cross section through spheroid showing variation of cell and nucleus radii according to their gaussian distribution. (C) Distribution of sampled cell and nucleus radii of MDA-MB-468 cell line.

**FIGURE 2.**
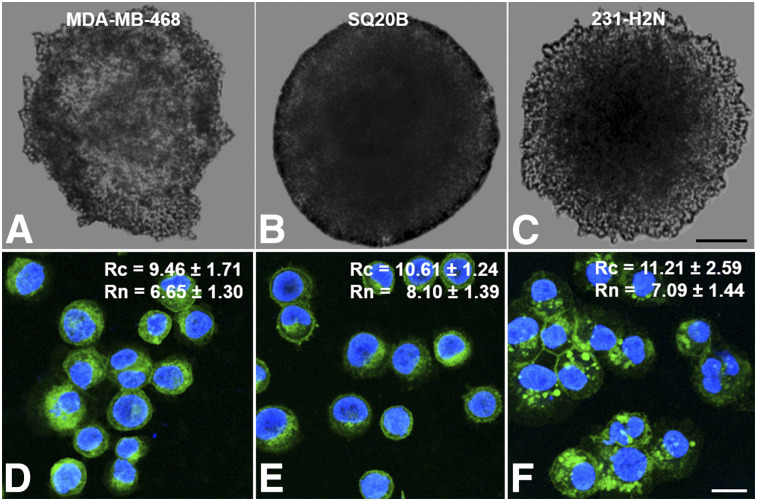
(A–C) Light microscopy images showing 3D spheroids of MDA-MB-468 (A), SQ20B (B), and 231-H2N (C) produced using hanging-droplet method (scale bar: 100 μm). (D–F) Confocal microscopy images of associated cell sizes, membrane (green: wheat germ agglutinin), and nuclei (blue: 4′,6-diamidino-2-phenylindole) for MDA-MB-468 (D), SQ20B (E), and 231-H2N (F) (scale bar: 20 μm). R_c_ = cell radius; R_n_ = nucleus radius.

A 3D rendering of an in silico MDA-MB-468 spheroid, a cross section through a smaller cluster (to demonstrate variation in cell and nucleus size), and the relationship between radii of cells and nuclei are shown in Figure 1. MDA-MB-468 spheroids comprising loosely associated cells were compared with 231-H2N and SQ20B spheroids (Figs. 2A–2C). SQ20B cells formed spheroids that had a rim of close-packed cells, with less closely packed cells toward the center. Cell and nucleus radii used for MC simulations were 9.45 ± 1.71 μm and 6.65 ± 1.30 μm, respectively, for MDA-MB-468, 10.61 ± 1.24 μm and 8.10 ± 1.39 μm, respectively, for SQ20B; and 11.21 ± 2.59 μm and 7.09 ± 1.44 μm, respectively, for 231-H2N (Figs. 2D–2F). The radius of each cell and corresponding nucleus were sampled from an empiric distribution of the cell line radii, considering the correlation between the two (Supplemental Fig. 1).

### Radioactivity Uptake and DNA Damage in Spheroids

#### ^111^In-EGF

**FIGURE 3.**
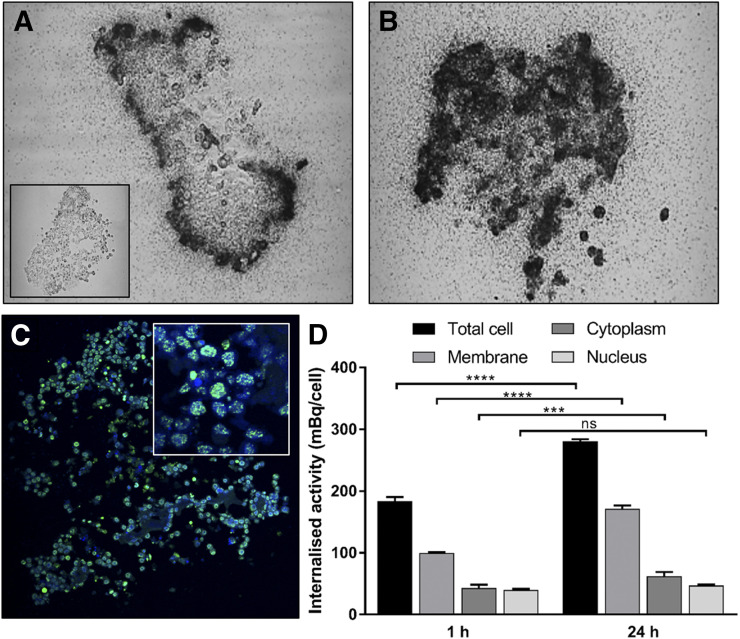
Spatial distribution of ^111^In-EGF in MDA-MB-468 spheroids. (A and B) Microautoradiograms of 8-μm spheroid sections after 1 h (inset shows control) (A) and 24 h (B) of treatment. (C) γH2AX expression in consecutive spheroid section after 24 h of treatment (inset shows uniform distribution of foci within cells throughout spheroid). (D) Internalized activity (mBq/cell) determined at 1 and 24 h of incubation. ***P < 0.0005. ****P < 0.0001. ns = not significant.

**FIGURE 4.**
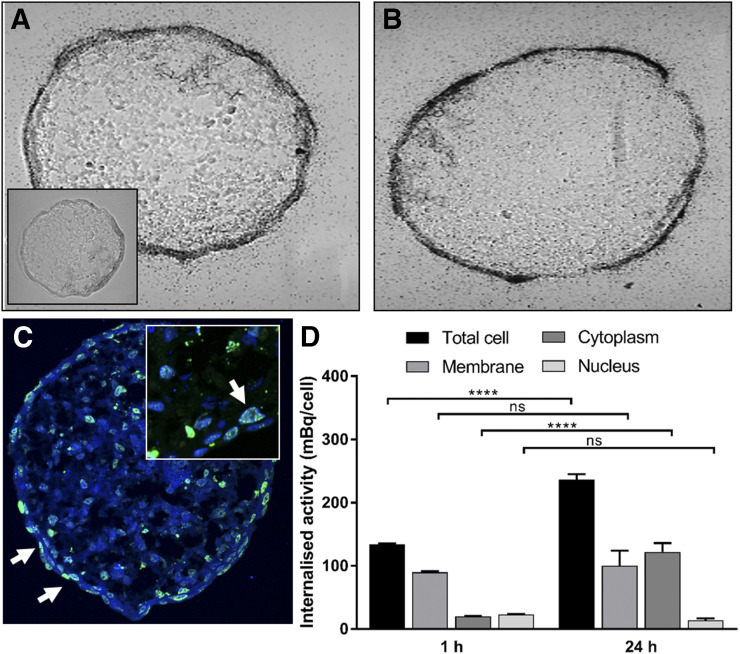
Spatial distribution of ^111^In-EGF in SQ20B spheroids. Microautoradiograms of 8-μm spheroid sections after 1 h (inset shows control) (A) and 24 h (B) of treatment. (C) γH2AX expression in consecutive spheroid section after 24 h of treatment (inset shows peripheral distribution of foci within spheroid). (D) Internalized activity (mBq/cell) determined at 1 and 24 h of incubation. ****P < 0.0001. ns = not significant.

At 1 h, ^111^In-EGF accumulated at the periphery of EGFR-positive MDA-MB-468 and SQ20B spheroids (Figs. 3A and 4A). Control untreated spheroids are shown for comparison (inset images, Figs. 3A and 4A). By 24 h, ^111^In-EGF had accumulated throughout MDA-MB-468 spheroids (Fig. 3B) whereas peripheral accumulation persisted in SQ20B spheroids (Fig. 4B). γH2AX induction reflected radioactivity distribution and at 24 h was observed throughout MDA-MB-468 spheroids (Fig. 3C) but was restricted to the periphery in SQ20B spheroids (Fig. 4C). The amount of ^111^In-EGF that had accumulated in whole cells and in membrane, cytoplasmic, and nuclear fractions by 1 and 24 h is shown for MDA-MB-468 cells (Fig. 3D) and SQ20B cells (Fig. 4D). In MDA-MB-468 cells, membrane and cytoplasmic fractions increased significantly with time (*P* < 0.0001). Nuclear accumulation remained virtually constant in SQ20B cells, whereas the cytoplasmic fraction increased significantly with time (*P* < 0.0001). EGFR-low 231-H2N cells showed minimal ^111^In-EGF internalization, whereas spheroids showed little microautoradiography (Supplemental Fig. 2) or γH2AX staining.

#### ^111^In-Tz

**FIGURE 5.**
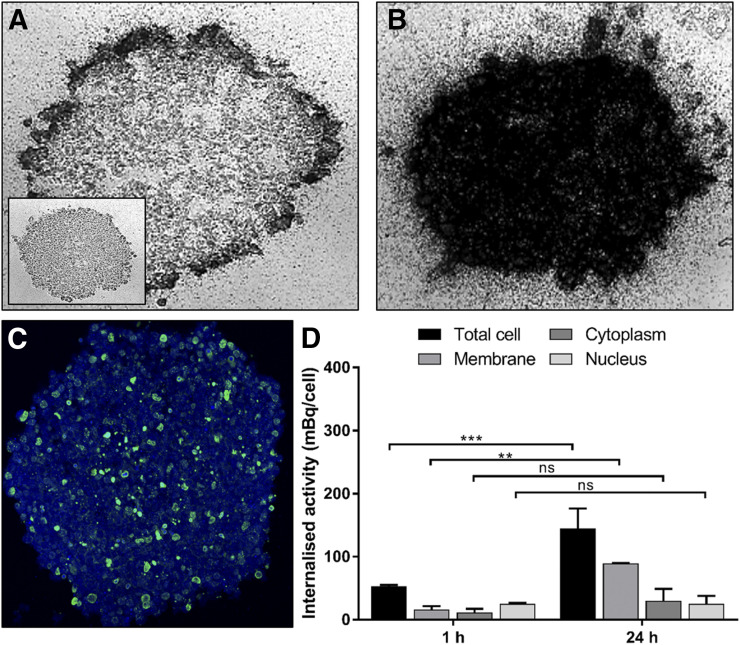
Spatial distribution of ^111^In-Tz in 231-H2N spheroids. Microautoradiograms of 8-μm spheroid sections after 1 h (inset shows control) (A) and 24 h (B) of treatment. (C) γH2AX expression in consecutive spheroid section after 24 h of treatment. (D) Internalized activity (mBq/cell) determined at 1 and 24 h of incubation. **P < 0.005. ***P < 0.0005. ns = not significant.

There was little microautoradiography staining or accumulation of ^111^In-Tz in the HER2-low cell lines MDA-MB-468 and SQ20B (Supplemental Fig. 3). Peripheral accumulation of radioactivity was seen after 1 h of incubation in HER2-high 231-H2N spheroids (Fig. 5A), with even distribution throughout the spheroid by 24 h (Fig. 5B). It is possible that the relatively sparse γH2AX staining in HER2-high 231-H2N spheroids (Fig. 5C), compared with MDA-MB-468 spheroids, despite uniform and marked accumulation of radioactivity throughout the whole spheroid could be explained by the relatively low nuclear accumulation of ^111^In in 231-H2N versus MDA-MB-468 cells.

### Clonogenic Survival

**FIGURE 6.**
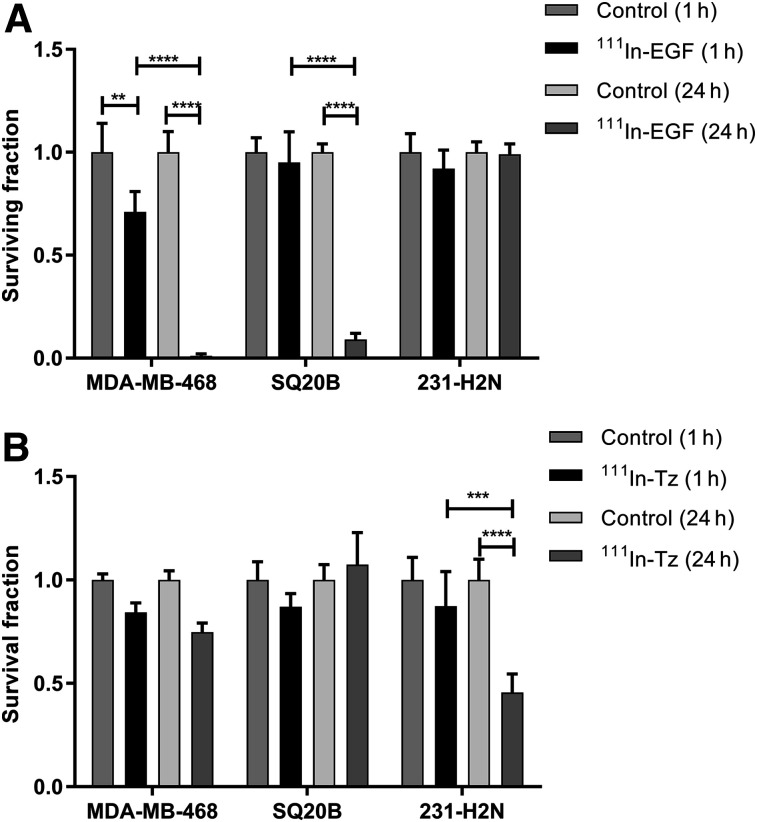
Clonogenic survival of MDA-MB-468, SQ20B, and 231-H2N cells after 1 or 24 h of treatment with ^111^In-EGF (8 MBq/μg, 40 nM) (A) or ^111^In-Tz (6 MBq/μg, 10 nM) (B). Error bars indicate SD of mean surviving fraction (*n* = 3). **P < 0.005. ***P < 0.0005. ****P < 0.0001.

After exposure to ^111^In-EGF, the surviving fraction of MDA-MB-468 cells decreased to 71% ± 10% after 1 h (*P* < 0.001) and to 1% ± 1% (*P* < 0.0001) after 24 h (Fig. 6A). For SQ20B cells, the surviving fraction was 9% ± 3% at 24 h (*P* < 0.0001). 231-H2N cells express the smallest number of EGFR and internalized the least activity, as reflected in the surviving fraction of 99% ± 5% at 24 h.

^111^In-Tz did not have a statistically significant effect on surviving fraction in the HER2-low cell lines MDA-MB-468 and SQ20B (Fig. 6B). However, cell survival in the HER2-positive cell line, 231-H2N, at 24 h was significantly different from that at 1 h (*P* < 0.0001), with the surviving fraction decreasing to 46% ± 9% at 24 h.

### MC Simulation

#### S Values

MC-calculated *S* values for single cells, monolayers, and cell clusters are summarized in Table 1. Single-cell *S* values agreed well with MIRDcell values, with differences of no more than 2%. RCP *S* values for monolayers and clusters take crossfire from neighboring cells into account. As a result of the variation in cell size and RCP, *S* values are given as an average value and SD. The greatest effect of crossfire was noted in cytoplasmic and cell-surface contributions, where S(N←Cy) increased by at least a factor of 2 in monolayers and almost a factor of 3 in spheroids across all cell lines, when compared with single-cell *S* values. In the case of 231-H2N, the S(N←Cs) contribution from RCP increased almost 5-fold compared with single cells. A graphical illustration of the contribution of cross dose to the total dose for cell clusters as a function of radial distance from the spheroid center is shown in Supplemental Figure 4, and the total contributions are summarized in Supplemental Table 1.

**TABLE 1:** Cellular *S* Values for Different Geometric Models and Cell Lines

Cell line	Single cells*			Monolayer^†^			Cluster – RCP^†^		
	S(N←N)	S(N←Cy)	S(N←Cs)	S(N←N)	S(N←Cy)	S(N←Cs)	S(N←N)	S(N←Cy)	S(N←Cs)
MDA-468	7.08E−04	8.47E−05	4.30E−05	9.83E−04 ± 7.36E−04	1.53E−04 ± 8.85E−05	1.04E−04 ± 5.86E−05	1.07E−03 ± 1.12E−03	1.94E−04 ± 7.63E−05	1.42E−04 ± 4.92E−05
SQ20B	4.21E−04	5.63E−05	3.21E−05	5.75E−04 ± 3.63E−04	1.01E−04 ± 2.84E−05	7.21E−05 ± 2.06E−05	7.10E−04 ± 8.30E−04	1.43E−04 ± 5.95E−05	1.09E−04 ± 3.93E−05
231-H2N	6.04E−04	5.35E−05	2.01E−05	1.01E−03 ± 1.33E−03	1.19E−04 ± 8.83E−05	6.98E−05 ± 5.66E−05	1.02E−03 ± 1.95E−03	1.45E−04 ± 1.04E−04	9.49E−05 ± 6.27E−05

* *S* values of each cell line are determined by MC simulation for average cell and nucleus radii given in Figures 2E–2F.

† Mean *S* value ± SD, for varied cell and nuclei radii (packing ratio for RCP ≈ 0.17).

Data are Gy/Bq s.

#### Surviving Fraction and TCP Evaluation

Cell compartment–specific uptake of the peptide and antibody constructs was evaluated to inform MC-calculated surviving fraction. Trapezoidal integration of the total activity at 1 and 24 h in the different cellular compartments provided the data necessary to calculate accumulated activity (*Ã*) (Table 2). Absorbed dose was then calculated (Eq. 2), and the calculated surviving fraction was determined using *S* values for 3D spheroids (Table 2). We assumed that the activity internalized in a single cell was representative of that in cells within a spheroid and that the experimental surviving fraction determined for monolayers was applicable to spheroids.

**TABLE 2:** Comparison of Surviving Fraction in ^111^In-Treated Tumor Spheroids

Treatment	Surviving fraction	MDA-468		SQ20B		231-H2N	
		1 h	24 h	1 h	24 h	1 h	24 h
^111^In-EGF	*Ã* cell (Bq s)*	331	19,558	242	15,594	13	1,533
	*Ã* nucleus (Bq s)	72	3,688	42	1,597	2	108
	Experimental^†^	0.71 ± 10	0.01 ± 1	0.95 ± 15	0.35 ± 12	0.92 ± 9	0.99 ± 5
	MC model 1^‡^	0.95	0.13	0.99	0.66	1.00	0.97
	MIRDcell^¶^	0.92	0.09	0.94	0.42	1.00	0.91
	MC model 2^§^	0.87	0.01	0.98	0.43	1.00	0.93
	MC model 3^g^	0.88	0.01	0.98	0.43	1.00	0.94
^111^In-Tz	*Ã* cell (Bq s)*	10	1,026	14	1,274	96	5,947
	*Ã* nucleus (Bq s)	1	67	2	170	46	1,884
	Experimental^†^	0.97 ± 5	0.75 ± 4	1.10 ± 6	1.08 ± 16	0.87 ± 17	0.46 ± 9
	MC model 1^‡^	1.00	0.92	1.00	0.96	0.99	0.67
	MIRDcell^¶^	1.00	0.89	1.00	0.90	0.98	0.69
	MC model 2^§^	1.00	0.84	1.00	0.91	0.97	0.19
	MC model 3^‖^	1.00	0.85	1.00	0.91	0.97	0.38

* Derived from mean whole-cell internalized activity using trapezoidal integration.

† Derived from technical triplicates.

‡ MC-derived surviving fraction based on α- and β-values determined from ^137^Cs irradiation.

¶ Calculated using mean cell or nucleus radius. α- and β-values from ^137^Cs irradiation are adopted for self-dose and cross dose.

§ Similar to MC model 1, but relative biological effectiveness of 4 is applied to dose deposited by radiation sources in nucleus (37,38).

‖ Similar to MC model 2, but Lea-Catcheside factor, G, is applied. G = 0.89 for cells incubated for 1 h. For cells incubated for 24 h, G for all cell lines is tabulated in Supplemental Table 2. All calculations assumed repair half-time of 1.5 h.

Experimental surviving fraction (Fig. 6) and calculated surviving fraction were compared with MIRDcell surviving fraction (Table 2) (12). MC model 1 and MIRDcell are both based on α- and β-values derived from ^137^Cs irradiation, the only difference being the packing algorithms used. MIRDcell assumes uniform cell and nucleus sizes in a close-packed cubic lattice. Across all cell lines, the 1-h surviving fraction data of MIRDcell and MC model 1 agreed well (<5% difference). However, MIRDcell surviving fraction was consistently lower (by ≤40%) at 24 h than MC model 1 surviving fraction. This finding was partly due to the higher cross-dose contribution from cubic lattice packing than from RCP and the variation in cell and nucleus size in the RCP cluster. If a threshold dose is required to kill a cell of a certain size, smaller cells will be killed but not bigger cells; with uniform cell sizes, all cells reaching that threshold dose will die. Neither model, however, adequately predicts the experimental surviving fraction. The second iteration, MC model 2, assumes a greater relative biological effectiveness when AE emitters accumulate in the nucleus of a cell. MC model 2 and experimental surviving fraction agreed well across all cell lines, apart from the 24-h surviving fraction data for the radioresistant cell line 231-H2N. This discrepancy can be attributed to the combination of the suppressed Lea-Catcheside factor, G (Supplemental Eq. 1), after 24 h of low–dose-rate exposure and radioresistance of the 231-H2N cell line (low α/β-ratio and high β). The next iteration, MC model 3, is similar to MC model 2 but also accounts for the suppression of G factor after exposure to ^111^In for 1 and 24 h. MC model 3 differed significantly only from MC model 2 for 231-H2N cells, and the surviving fractions derived using this model agreed well with the experimental surviving fraction.

**FIGURE 7.**
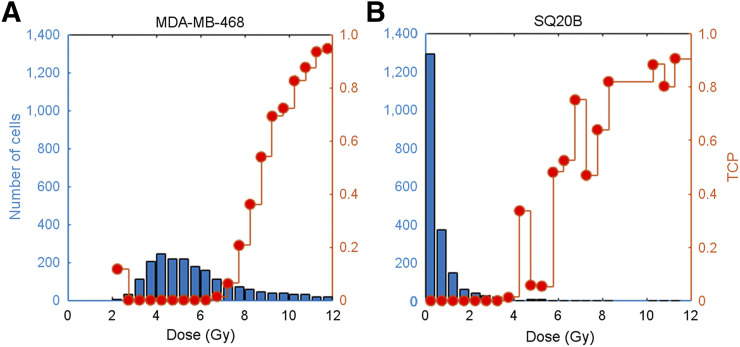
MC-simulated dose histograms of MDA-MB-468 (A) and SQ20B (B) spheroids after 24 h of exposure to ^111^In-EGF and TCP for each dose bin of 0.5 Gy (right vertical axis).

When radioactivity was restricted to the periphery of the spheroids, the calculated TCP became zero. If physical decay alone was considered, and no further internalization of ^111^In-conjugated constructs was allowed after 24 h, only MDA-MB-468 spheroids exposed to ^111^In-EGF achieved a TCP of 50% for all 3 MC models (after 5.3, 1.9, and 2 d for models 1, 2, and 3, respectively). This is evident from Figure 7, where unlabeled cells receive negligible doses due to the weak crossfire effect associated with ^111^In.

## DISCUSSION

Here, the efficacy of a peptide and antibody delivery strategy for AE-targeted radiotherapy was evaluated in spheroid models of 3 different cell lines, namely MDA-MB-468, SQ20B, and 231-H2N. Spheroid morphology was then used to generate 3D geometry models for MC simulation to predict TCP using data from activity distribution, internalization, and survival assays to build realistic in silico models.

It was immediately evident that spheroid morphology differed among cell lines. MDA-MB-468 cells did not form compact round spheroids as did the other breast cancer cell line, 231-H2N (Fig. 2), but, rather, formed loose aggregations, a characteristic previously observed by Ivascu et al. (6). This loose aggregation of cells facilitated the penetration of the ^111^In-labeled peptide as seen on microautoradiography images, which showed a large accumulation of silver grains on the rim of MDA-MB-468 spheroids after a 1-h incubation with ^111^In-EGF, spreading over the whole spheroid section after incubation for 24 h (Figs. 3A and 3B). In contrast, SQ20B spheroids consisted of closely packed cells at the periphery, compared with the core. It is possible that the dense outermost cell layers established a diffusion barrier, which prohibited the penetration of the radiopeptide. Microautoradiography results confirmed that the accumulation of silver grains was restricted mostly to the rim of the spheroid at 1 and 24 h (Figs. 4A and 4B). The EGFR-low cell line, 231-H2N, showed little positive microautoradiography staining at either time point (Supplemental Fig. 3). The situation was reversed when we evaluated the spatial distribution of the ^111^In-labeled antibody, ^111^In-Tz. The HER2-low cell lines, MDA-MB-468 and SQ20B, showed barely any microautoradiography staining at 1 or 24 h (Supplemental Fig. 4) whereas ^111^In-Tz–treated 231-H2N spheroids showed a peripheral staining pattern at 1 h that spread throughout the spheroid by 24 h (Figs. 5A and 5B).

Microautoradiography staining, although informative, does not account for the nonuniform intracellular radionuclide distribution, which could significantly alter the energy deposition in the cell (or cell nucleus), especially in the context of short-range electron emission (13). Hence, microautoradiography staining was used to provide a global representation of activity distribution, whereas internalization assays provided the activity distribution at a subcellular level. To confirm that activity distribution correlated with DNA damage, γH2AX staining was performed. γH2AX staining patterns for ^111^In-EGF–treated MDA-MB-468 and SQ20B spheroids after 24 h correlated well with microautoradiography, showing γH2AX induction throughout the MDA-MB-468 spheroid and peripheral induction in only the SQ20B spheroid. There was less γH2AX induction in SQ20B cells than in MDA-MB-468 cells, as the latter accumulated almost twice the nuclear activity. With ^111^In-Tz–treated 231-H2N spheroids, γH2AX foci (Fig. 5C) were sparsely distributed compared with ^111^In-EGF–treated MDA-MB-468 spheroids (Fig. 3C), a finding that initially was surprising given the uniform microautoradiography staining in both spheroid models. On closer inspection, it was clear from internalization data that most of the activity was bound to the surface of 231-H2N cells, as would explain the less abundant γH2AX staining.

To relate accumulated activity to cell survival after 1 and 24 h of treatment with the ^111^In-labeled peptide and antibody constructs, clonogenic assays were performed using cells grown in monolayers (Fig. 6). An assumption made here was that surviving fractions measured in monolayers could be extrapolated to 3D spheroids given the same subcellular distribution of activity. It would of course be more informative to use treated spheroids for clonogenic assays; however, in our hands disaggregation of spheroids after radionuclide treatment led to significant cell loss, with the result that colony-counting experiments were not reproducible. Rae and Mairs (31) recently discussed the utility of using a multicellular spheroid growth assay as an alternative measure of clonogenicity. However, because our endpoint was to determine TCP of the different AE-treatment strategies, estimation of clonogenic surviving fraction was necessary. These data were then used with MC-calculated *S* values to estimate absorbed dose. In addition, these data were compared with MIRDcell data. The MIRDcell software provides estimates of *S* values in single cells, cell monolayers, and cells clustered in a closely packed geometry and predicts the surviving fraction of targeted and nontargeted cell populations in the last of these (12). Although close-packed hexagonal or cubic lattices as used by MIRDcell are mathematically simpler models than RCP algorithms, they do not reflect the disorganized cellular architecture of tumors, nor do they account for the nonuniform distribution of cell size and nucleus size (32,33). *S* values are greatly affected by cell volume; thus, *S* values (and hence dose) may be significantly under- or overestimated when variation in cell volume is ignored. Furthermore, activity is often assumed to be homogeneously distributed to simplify simulation. Here we have determined the intracellular distribution of activity in individual cells, as well as the spatial distribution by microautoradiography in tumor spheroids. By incorporating the experimentally determined radioactivity distribution with robust MC simulation, it is possible to construct models to predict radiobiologic endpoints. For this reason, monodisperse spheric RCP cells reflecting the variation in cell and nucleus radius and radioactivity distribution were used to generate monolayer and 3D MC spheroid models.

When we compared the experimental results with the MC model 1 surviving fraction after 1 h incubation with ^111^In-EGF (Table 2), we noted differences of up to 33% for MDA-MB-468. In contrast, Cai et al. (14) reported differences of less than 4% between MC-calculated and experimental results. The main factors contributing to these discrepancies can be ascribed to the bigger cell radii reported in this paper and activity estimation, which was determined by trapezoidal integration in this instance and not assuming instantaneous uptake. When these 2 factors are considered, calculated surviving fraction does not adequately predict experimental surviving fraction.

One aspect that we have not addressed is the adoption of radiosensitivity parameters for the different cell lines generated by ^137^Cs irradiation. With a protracted radiation delivery, as is the case with targeted radionuclide therapy, radiation repair will occur and the Lea-Catcheside factor can no longer be assumed to be 1 (Supplemental Table 2). Furthermore, the α-component from high–dose-rate ^137^Cs irradiation is likely to overestimate the radiosensitivity compared with internalized ^111^In apart from the scenario in which ^111^In is incorporated or close to the DNA in the nucleus. There is a difference in radiotoxicity arising from self-irradiation and cross irradiation, especially with AE-emitting radionuclides associated with the DNA (34). To account for this difference, MC model 2 incorporated relative biological effectiveness–adjusted α/β ratios for nucleus-associated AE emitters, with a subsequent good correlation with experimental surviving fractions. A further iteration, MC model 3, which took repair into consideration, resulted in an even better prediction of experimental surviving fraction. However, TCP probability estimates (Fig. 7) clearly show that only those cells with a high enough nuclear activity uptake will accumulate a lethal dose; neighboring cells will be spared (15,35).

Considering that spheroids are representative of micrometastases in their prevascular stage of development, the results observed here confirm that the single most important aspect determining the biological efficacy of an AE-emitting targeting strategy is uniform nuclear accumulation of the construct. Even though ^111^In-Tz was uniformly distributed throughout the 231-H2N spheroid, TCP was not achieved, as a result of limited nuclear accumulation. Similarly, whereas EGFR expression level was similar in SQ20B and MDA-MB-468 cells, the nuclear accumulation of ^111^In-EGF was much less for SQ20B than for MDA-MB-468. Also, the 3D morphology of the spheroid prohibited adequate penetration of ^111^In-EGF, resulting in a negligible TCP. Given these results, it is plausible that an AE-emitting targeted radionuclide therapy agent could be used in a clinical scenario to target limited-volume disseminated cancer and micrometastases. Indeed, in the first-in-humans trial using ^111^In-EGF tumor, localization was achieved without any serious adverse effects (36).

## CONCLUSION

It is essential, when evaluating the distribution of radionuclides in models of micrometastatic disease, to include biologic endpoints such as TCP. As shown here, the spatial distribution of radioactivity at subcellular and multicellular levels is a clear indicator of biological effect and TCP.

## DISCLOSURE

This work was supported by grants from Cancer Research U.K. (CRUK) (C5255/A15935), the Medical Research Council (MRC) (MC-PC-12004), and the CRUK Oxford Centre. No other potential conflict of interest relevant to this article was reported.
